# Efficacy of Metformin for Benign Thyroid Nodules in Subjects With Insulin Resistance: A Systematic Review and Meta-Analysis

**DOI:** 10.3389/fendo.2018.00494

**Published:** 2018-08-28

**Authors:** Miao Sui, Yuan Yu, Huifeng Zhang, Hongjie Di, Chao Liu, Yaofu Fan

**Affiliations:** ^1^Endocrinology Laboratory, The Third Clinical Medical College, Nanjing University of Chinese Medicine, Nanjing, China; ^2^Department of Endocrinology, Xuzhou Hospital of Traditional Chinese Medicine, Xuzhou, China; ^3^Department of Gastroenterology, Jiangning District Hospital of Traditional Chinese Medicine, Nanjing, China; ^4^Endocrine and Diabetes Center, Jiangsu Province Hospital on Integration of Chinese and Western Medicine, Nanjing University of Chinese Medicine, Nanjing, China

**Keywords:** benign thyroid nodule, thyroid nodules volume, metformin, insulin resistance, meta-analysis

## Abstract

**Background:** To evaluate the effect of metformin therapy on decreasing benign thyroid nodule volume in subjects with insulin resistance (IR).

**Method:** Randomized controlled trials (RCTs) and self-controlled trials for the meta-analysis published, before January 31, 2018 were selected from the PubMed, Cochrane Library, Embase, Web of Science, Chinese Biomedical Literature Database, National Knowledge Infrastructure, WANFANG and VIP Database. Pooled standard mean difference with 95% confidence interval was estimated by fixed- or random-effects model depending on heterogeneity. The risk of bias using the Cochrane Collaboration's tool was used to assess the quality of the RCTs contained. The quality of self-controlled studies was evaluated using the Methodological index for non-randomized studies (MINORS) method.

**Results:** 7 studies (3 RCTs and 4 prospective self-controlled studies) with 240 patients were considered to be appropriate for the meta-analysis. The results of the meta-analysis indicated that the volume of thyroid nodule decreased significantly after metformin therapy (SMD −0.62, 95% CI −0.98 ~ −0.27). 6 studies reported the changes of the level of TSH. TSH levels decreased significantly after metformin therapy (SMD −0.27, 95% CI −0.47 ~ −0.07). The pooled data indicated an increase in FT3 level, and an unchanged FT4 level after metformin therapy (FT3, SMD 0.25, 95% CI 0.05 ~ 0.45; FT4, SMD −0.07, 95% CI −0.27 ~ 0.13). HOMA-IR levels decreased significantly after metformin therapy based on the pooled results of 3 RCTs and 3 prospective self-controlled studies (SMD −1.08, 95% CI −1.69 ~ −0.47).

**Conclusion:** The meta-analysis demonstrated that metformin was safe and useful in shrinking benign thyroid nodules volume, improving thyroid function and IR. A large number of high-quality prospective studies still need to be carried out.

## Introduction

Lately, there has been increased interest in the effects of insulin resistance (IR) on the thyroid, several studies have reported an association between IR and thyroid nodules (1–3). IR is a risk factor of hypertension, abdominal obesity, impaired glucose metabolism, nonalcoholic fatty liver disease, and other disorders (4, 5). Various studies have implicated chronic activation of the pro-inflammatory pathway can be a mechanism for IR (6). It has been shown that insulin-like growth factor (IGF)-1 and its receptor and insulin receptor was found in thyroid cancer, suggesting that insulin receptor overexpression may play a role in the etiopathogenesis of thyroid tumorigenesis (7, 8). International Diabetes Federation (IDF) announced a continual increase in the rate of diabetes around the world. Similarly, the incidence of thyroid nodule was also rising. One of the characteristics of type 2 diabetes mellitus (T2DM) patients is the presence of IR and the response of hyperinsulinemia. Insulin is known to act as a growth factor that stimulates cell proliferation. In some reports, greater thyroid volume and higher nodule prevalence were observed in patients with T2DM patients and thyroid volume was positively correlated with hyperglycemia in patients with impaired glucose metabolism (3, 9). However, few studies have focused on the relationship between thyroid nodules and IR in patients with T2DM.

Thyroid nodule is a common worldwide thyroid disorder, with an estimated prevalence of 4–7% via palpation, whereas it can be identified by US in 10–41% of the individuals (10). Most of the Thyroid nodules are benign (noncancerous), and only less than 10% are malignant (11). Although the treatment of thyroid nodule is often not warranted, some patients still need the treatment due to pressure symptoms or cosmetic complaints (12). Treatment options for the benign thyroid nodules include observation, surgery, radioiodine therapy, and thyroid hormone suppression therapy. All these treatment options have several drawbacks.

Metformin is an antidiabetic drug, which is frequently prescribed and recommended to patients with IR. Recently, a few studies revealed the potential effects of metformin on treating thyroid-related characteristics, such as inhibitory effect of metformin on the growth of human thyroid cells (13), and thyroid cancer (14). However, the effect of metformin on benign thyroid nodules in subjects with IR remains unclear. Recently, a couple of randomized controlled trials (RCTs) and non-randomized controlled trials were performed to estimate the association between IR and thyroid nodule, but no relevant meta-analyses have been conducted. Here, this systematic review and meta-analysis was performed to evaluate the effectiveness of metformin on benign thyroid nodules in subjects with IR.

## Methods

This meta-analysis was conducted on the basis of the Preferred Reporting Items for Systematic Reviews and Meta-Analyses (PRISMA) guidelines (15). Whether ethical or patient written informed consent is necessary, as a result of systematic reviews and meta-analysis based on published studies. Two of independent reviewers performed all the steps and discrepancies were solved by consensus with a third author.

### Search strategy

The comprehensive literature searches were independently conducted by 2 individual investigators (Sui M and Yu Y) with the same method in PubMed, Cochrane Library, Embase, Web of Science, Chinese Biomedical Literature Database, National Knowledge Infrastructure, WANFANG and VIP Database until January 31, 2018. We used the following search terms in the field for Title/Abstract and/or keywords: “metformin” or “biguanide” or “dimethylbiguanide” and “thyroid nodule” or “thyroid goiter.” In addition, we conducted a manual search on the reference lists of the retrieved articles, reviews and trial registration databases to identify items missed by the electronic search. If multiple studies of the same patient population were identified, we only included the published report of the largest sample size.

### Study selection

Articles were included in our study if they met the following criteria: included randomized and non-randomized controlled trials, prospective and retrospective cohort studies, and case-control studies discussing the association between metformin and thyroid nodule with IR; clinical results, such as thyroid volume, nodule volume, thyroid-stimulating hormone (TSH), free tri-iodothyronine (FT3), free tetra-iodothyronine (FT4), and homeostasis model assessment (HOMA) index.

Studies met exclusion criteria if they were abstracts, case reports, case series, *in vitro* studies, and animal studies; did not use ultrasound to evaluate nodule volume; the studies with malignant thyroid nodule; studies using non-categorical data.

After obtaining a full list of studies, the same reviewers independently assessed each study for eligibility for inclusion in the meta-analysis, and data were extracted according to the study criteria and entered into a spread sheet. Differences between reviewers were resolved by discussion, and an agreement on the final dataset was reached by consensus.

### Data extraction

Two investigators (Sui M and Yu Y) independently extracted the following information and entered them into a data extraction database: authors, year of publication, study design, interventions, male or female number, age, the number of treatment sessions, outcomes (including percentage mean change [absolute mean change (mL)] in thyroid volume and nodule volume, TSH, FT3, FT4 and HOMA-IR levels). If critical data were unavailable in the articles, we contacted the original authors to request them.

### Assessment of study quality

The risk of bias using the Cochrane Collaboration's tool was performed to assess the quality of the included RCTs (16). It contains 7 bias metrics including selection bias (random sequence generation and allocation concealment), performance bias (blinding of participants and personnel), detection bias (blinding of outcome assessment), attrition bias (incomplete outcome data), reporting bias (selective reporting), and other bias. During data extraction and quality assessment, any disagreement between the two investigators was settled by discussion with a third reviewer (Zhang HF) to reach a consensus.

The quality of self-controlled studies was evaluated by the Methodological index for non-randomized studies (MINORS) method (17), based on the following items: a stated aim of the study; inclusion of consecutive patients; prospective collection of data; endpoint appropriate to the study aim; unbiased evaluation of endpoint; follow-up period appropriate to the major endpoint; and loss to follow-up not exceeding 5%.

### Statistical analysis

All statistical tests for this meta-analysis were performed with Stata 12.0 software. However, “risk of bias” assessment was realized by employing the RevMan 5.2 software. Odds ratios (ORs) with 95% of confidence interval (CIs) were calculated to assess the effect of dichotomous data. *I* ^2^ and *P* values were calculated to assess the heterogeneity among studies (*I* ^2^ > 50% and/or *P* < 0.1 were considered to be statistically significant). If there was no statistical difference in heterogeneity (*P* ≥ 0.05), the assumption of homogeneity was deemed valid and a fixed-effect model was then applied. Otherwise, a random-effects model would be used (18). Sensitivity analysis was performed on our results to assess the potential impact of various biases on our results. The risk of publication bias of included studies was checked by the visual inspection of symmetry level of funnel plot and Egger linear regression test (19). All statistical tests were two sided. A *P-*value less than 0.05 was considered to be statistically significant.

## Results

### Study selection

**Figure 1:**
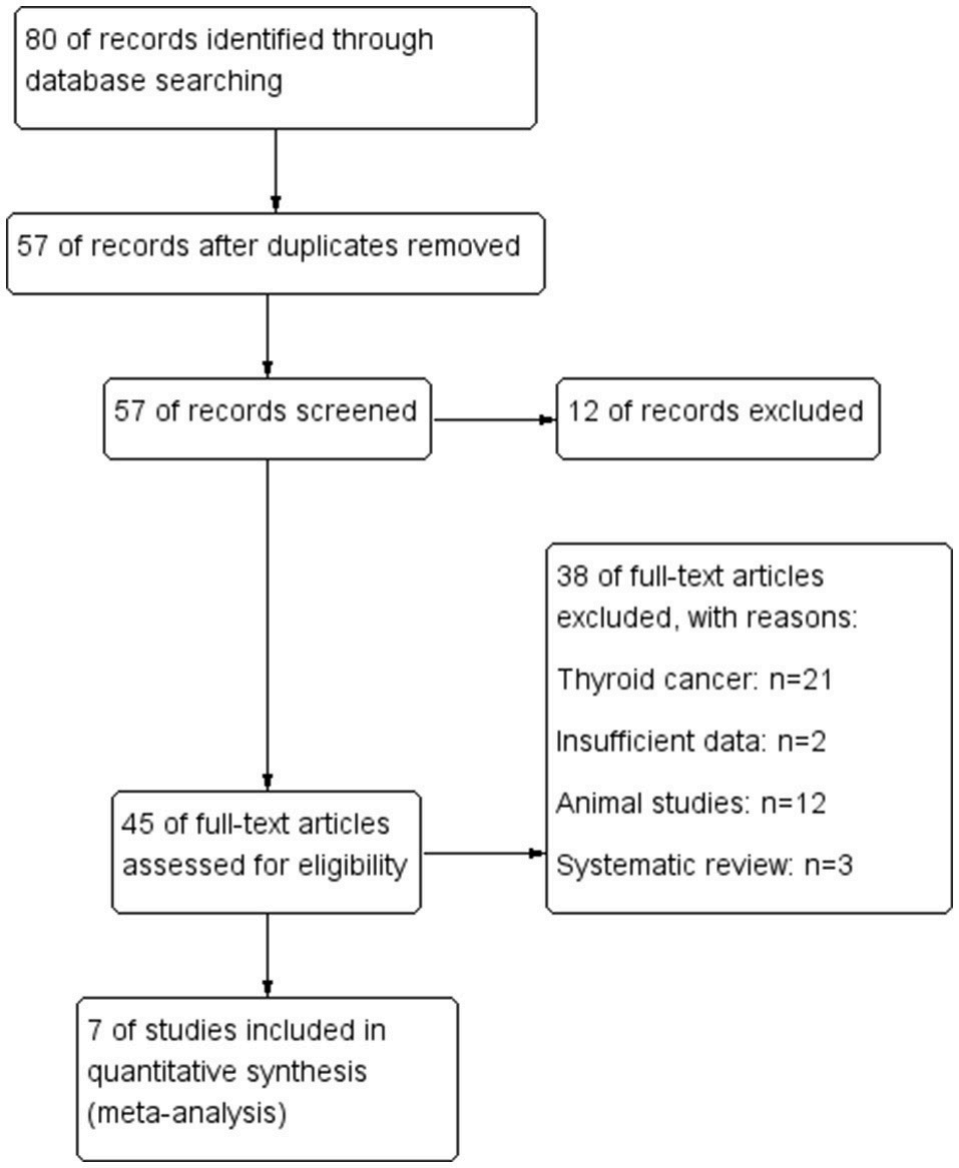
Flow diagram of the study selection process.

Our initial search identified 36 references in English, and 44 references gained from Chinese databases according to the criterion for study selection. Then, after browsing the title, the scope of the records was narrowed. Finally, 7 studies (20–26) meet the inclusion criteria and were selected for our study (Figure 1).

### Baseline characteristics and study quality

**Figure 2:**
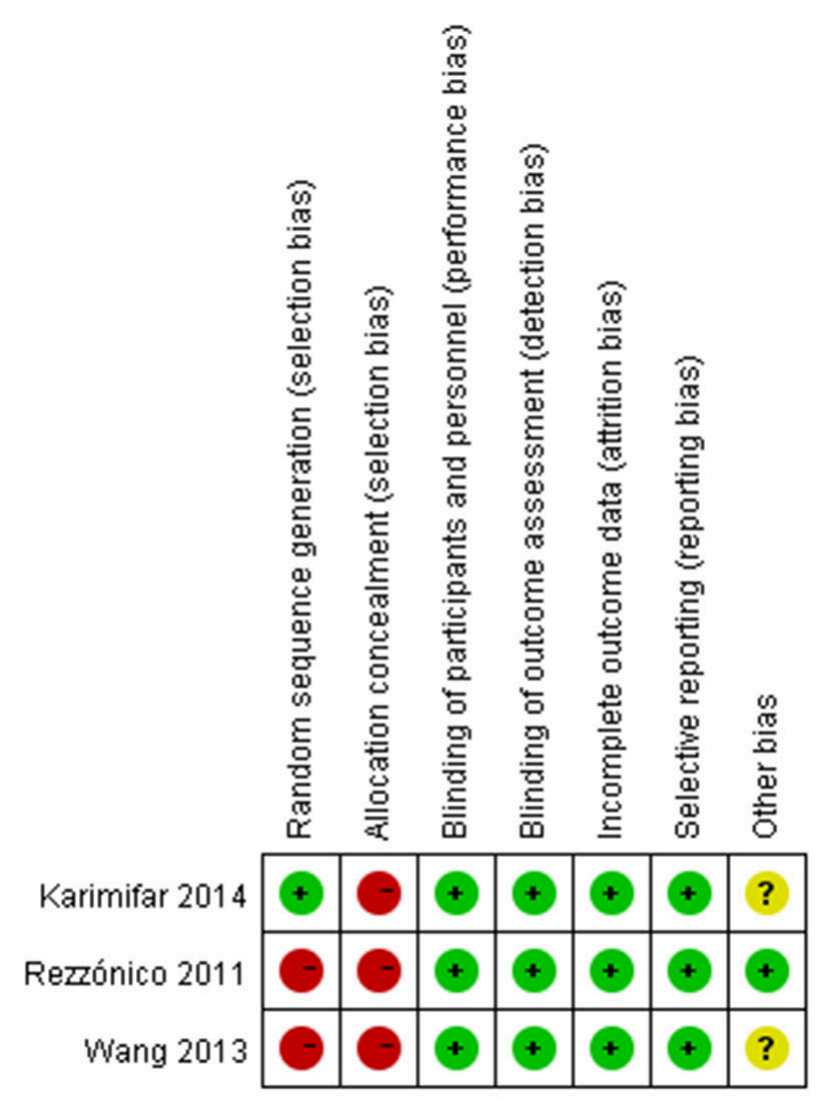
Risk of bias summary: review authors' judgments about each risk of bias item for each RCTs. –, high risk; +, low risk; ?, unclear risk.

**Figure 3:**
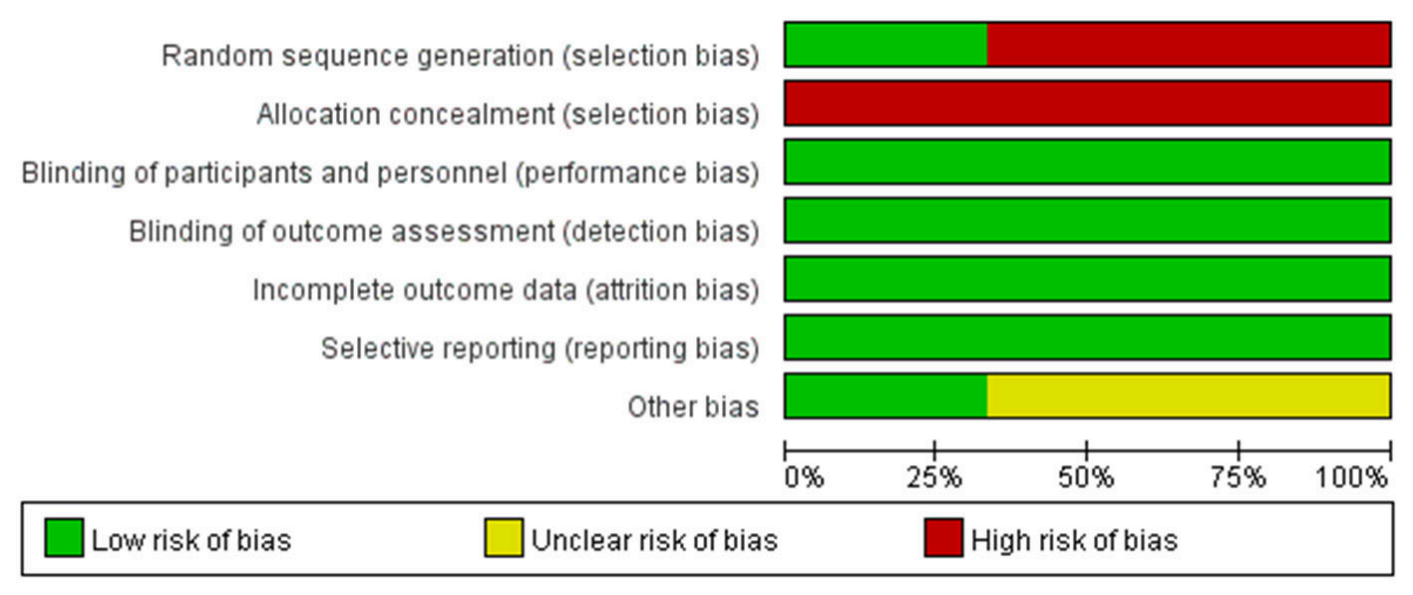
Risk of bias graph: review authors' judgments about each risk of bias item presented as percentages across all included RCTs.

The main characteristics of the studies were shown in Table 1. A total of 240 subjects from the 7 studies were included in the meta-analysis with a 3–12 months range of treatment duration. We used MINORS to assess the quality of the self-controlled trials (SCTs). In general, the included self-controlled trials were of moderate quality (Table 1). Based on the investigators' judgment on the risk of each RCT's bias project, a summary and chart of the bias risk was constructed and used as a percentage of all the studies included (Figures 2, 3).

**Table 1:** Characteristics of Studies included in the meta-analysis.

**Study (References)**	**Year**	**Region**	**Male/Female**	**Mean Age, y**	**Meformin Dosage, mg/d**	**Duration (mon)**	**Quality score**
Rezzónico et al. 20	2011	Argentina	NR	46.4 ± 2.42	2,000	6	12
Wang 21	2013	China	0/14	46.4 ± 2.4	1,500	6	10
Karimifar et al.^22^	2014	Iran	39/4	51.1 ± 1.22	NR	3	12
Wei et al. 23	2015	China	8/10	60 ± 7	1,500–2,000	12	10
Anil et al. 24	2016	Turkey	32/68	52.5 ± 10.3	1,700	6	11
Cao 25	2017	China	12/9	42.5 ± 3.3	1,500	6	9
Zhang 26	2017	China	18/12	51.5 ± 11.2	1,500	6	9

### Volume of thyroid nodule

**Figure 4:**
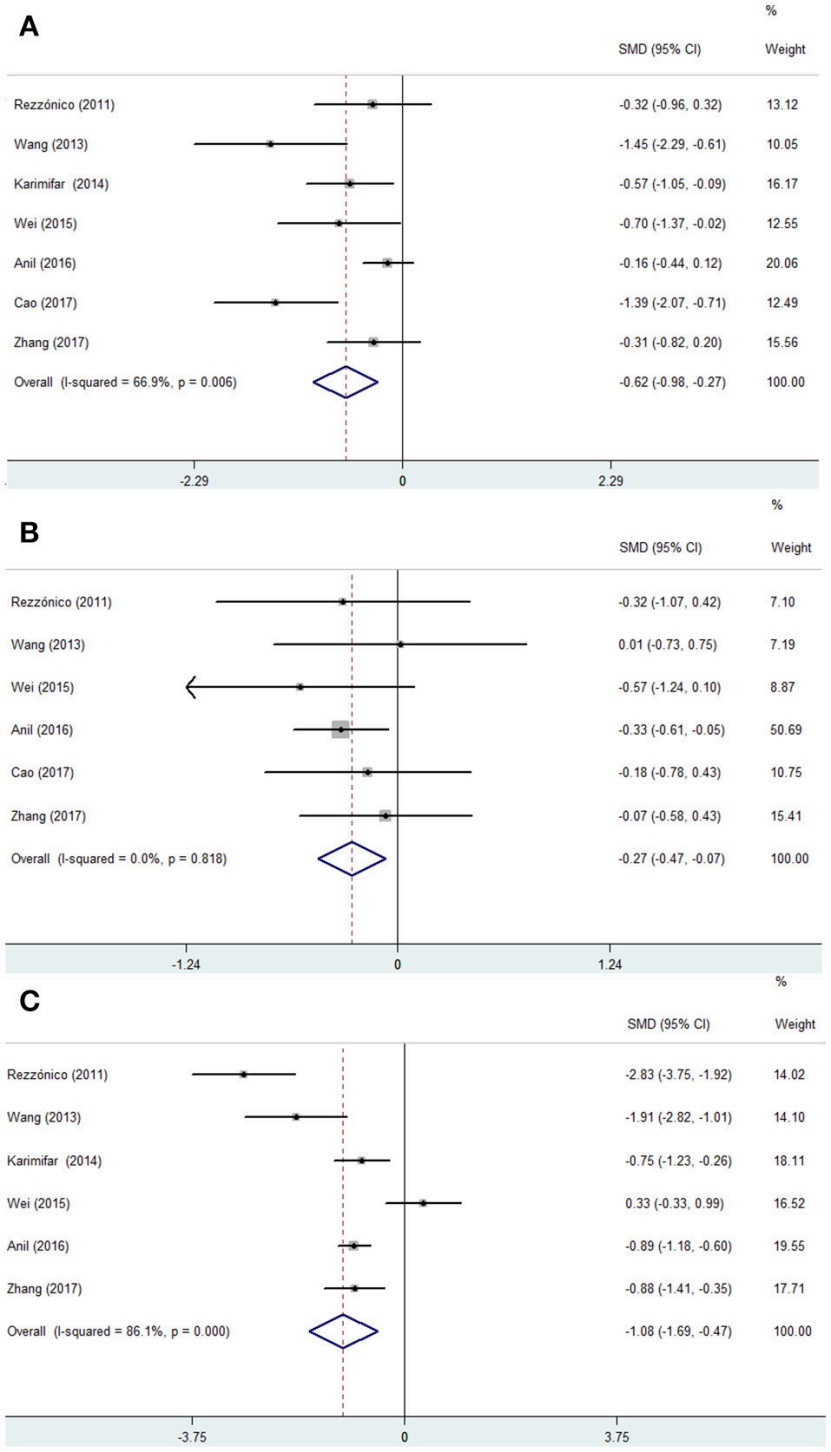
Forest plots from a meta-analysis of the effects of metformin on the thyroid nodule size **(A)** and the level of TSH **(B)** and HOMA-IR **(C)**.

In pooled results from 7 studies [3 RCTs (20–22) and 4 prospective self-controlled studies (23–26)], the volume of thyroid nodule decreased significantly after metformin therapy (SMD −0.62, 95% CI −0.98 ~ −0.27) (Figure 4A). Meanwhile, significant heterogeneity was found across the studies. To determine the source of heterogeneity, we conducted a sensitivity analysis and found results from the study by Anil et al. (24) were the causal. Because the large sample size in this study might cause instability for our the results, we included their data using the random-effect model.

### Thyroid function

Six studies [2 RCTs (20, 21) and 4 prospective self-controlled studies (23–26)] reported the changes of TSH level, which decreased significantly after metformin therapy (SMD −0.27, 95% CI −0.47 ~ −0.07) (Figure 4B). Meanwhile, the pooled data also indicated an increase in FT3 levels, but an unchanged FT4 levels after metformin therapy (FT3, SMD 0.25, 95% CI 0.05 ~ 0.45; FT4, SMD −0.07, 95% CI −0.27 ~ 0.13). Sensitivity analysis of 3 models showed robust results with no heterogeneity among studies (*I*^2^ = 0) (figure not shown), so the fixed-effect model was applied.

### Efficacy of metformin for HOMA-IR

HOMA-IR levels decreased significantly after metformin therapy based on the pooled results of 3 RCTs and 3 prospective self-controlled studies (SMD −1.08, 95% CI −1.69 ~ −0.47) (Figure 4C). Sensitivity analysis showed that the result was not robust.

### Publication bias

**Figure 5:**
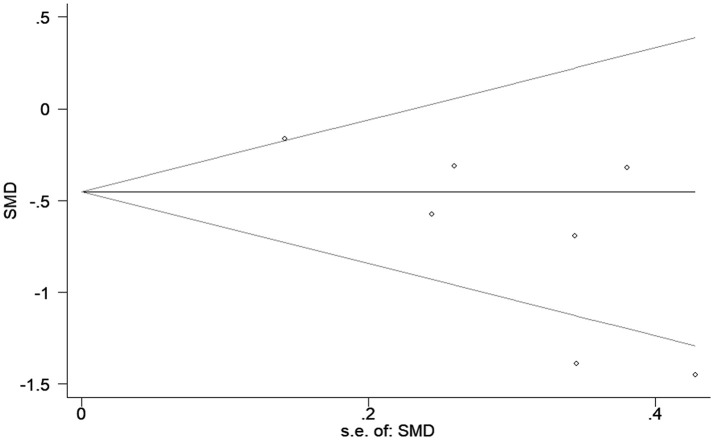
Begg's funnel plot of included studies for potential publication bias. SMD, standardized mean differences; s.e., standard error.

The funnel shape of the plot was not perfectly symmetrical, indicating a potential publication bias. Although we conducted comprehensive searches for avoiding bias to the greatest extent, 4 out of the 7 studies were published in China, and accordingly, we could not exclude potential publication bias (Figure 5).

## Discussion

To the best of our knowledge, this is the first meta-analysis to evaluate the effectiveness of metformin for benign thyroid nodules in subjects with IR. The data of our analysis indicated a decrease in nodule volume, TSH level, and HOMA-IR level, an unchanged FT4, and an increased FT3 level after metformin therapy over the short term (3–12 months). However, in this meta-analysis, changes in serum FT3 levels were not consistent with changes in serum T4 levels. Our analysis showed that heterogeneity existed across several of the evaluated studies, so we conducted sensitivity analysis in these cases, and the results suggested the heterogeneity did not affect the final results.

The global prevalence of diabetes is predicted to be 11.1% in 2033, impacting around 600 million people (27). Numerous epidemiological studies manifest the higher prevalence of thyroid disease in T2DM population than in the general population (3, 28). Smoking, IR, obesity, and diet preference have been widely investigated among patients with T2DM and thyroid nodule (29). In a study by Heidari et al. higher HOMA-IR values were found in patients with thyroid nodules and the values were associated with benign thyroid nodules (30). In another study, ayturk and colleagues showed that patients with IR have a significant increased thyroid volume and nodule prevalence (31). IR increases visceral fat accumulation which in further enhances TSH secretion (32). TSH and IGF-1 work synergistically to induce thyrocyte proliferation (33). Therefore, alleviating the IR may alleviate thyroid nodule growth rate as well as its volume and size. Metformin, an oral antidiabetic compound, is regarded as a first-line drug for treatment of T2DM with IR. Its pleiotropic effects mediated via AMP-activated kinase, lead to decreased fatty acid oxidation, inhibition of the proinflammatory pathway in perivascular adipose tissue, improving insulin resistance condition. It cannot be excluded that metformin has a direct, antiproliferative effect on the thyroid, possibly via suppressing hypothalamic-pituitary-thyroid axis activity, which might contribute to these observed effects (34).

In the current study, we found increased FT3 level, and reductions in TSH level and thyroid nodule volume after metformin therapy, but without change in FT4 level. The reduced serum T3 level was inconsistent with unchanged serum T4 level in this meta-analysis. Consistently, Cappelli et al. also found that one year metformin treatment unchanged FT4 levels in any group in that study (35). At first glance, it seems clear that TSH is the major growth factor of the thyroid gland. Nevertheless, suppressing TSH for a long term so as to decrease the size or arrest the growth of thyroid nodules is still highly controversial (36). A beneficial effect of thyroid hormones on diffuse goiters has been demonstrated in several controlled trials (37–39). Metformin may alter the affinity and/or expression levels of thyroid hormone receptors, increase dopaminergic tone, or induce constitutive activation of the TSH receptor (40). However, the mechanisms by which metformin would lower serum TSH levels are still debated. Cappelli et al. indicate that the TSH-lowering effect of metformin appears to occur independently of the presence of antithyroid peroxidase autoantibody (41). In a retrospective but large cohort of euthyroid diabetic patients, Díez and Iglesias (42) did not find any significant relationship between TSH and metformin treatment. Cappelli et al. showed that metformin treatment unchanged TSH level in euthyroid diabetic patients in that study (35). Our result reported significant TSH reduction with metformin in patients. In another clinical trial, Rotondi et al. demonstrated that metformin treatment lowered-TSH in hypothyroid patients regardless of levothyroxine usage (43). In addition, long-term treatment with metformin may regulate the leptin mRNA expression, and leptin is linked to hypothalamic-pituitary thyroid axis, which can regulate the TRH and TSH via JAK-2 signal transducer (44). So, we consider that the impact of metformin on thyrotrope function is partially associated with the changes in dopaminergic regulation of thyrotropin secretion (35, 45), but it still needs fully elucidating by further studies.

The meta-analysis also has several limitations to be mentioned. The sample size of the study is generally small, this may affect the validity of the statistical test. Some negative or irrelevance results related to metformin treatment for thyroid nodule volume might not be published. All these missing data and the limited number of studies hampered the execution of subgroup analyses, affecting our assessment of management of thyroid nodules after metformin. Undeniably, residual confounding effects between included studies cannot be excluded absolutely. The dosage of metformin and duration of included studies were different, which may lead to certain bias. At the same time, given the limitation of the researchers' language ability, more Chinese articles were included, which could lead to a certain selection bias. Finally, a part of published articles enrolled in our study were with poor quality. The lack of long-term follow-up data also limits our understanding of the efficacy and safety of metformin.

## Conclusion

In summary, the pooled meta-analysis of included studies demonstrated significant differences in thyroid nodule volume, TSH, FT3, and HOMA-IR between before and after metformin treatment for benign thyroid nodules in subjects with IR. Nevertheless, high quality prospective studies are needed for the application of metformin in the treatment of thyroid nodules.

## Author contributions

YF and CL conceived and designed the research and critically revised the manuscript. HZ and HD drafted the protocol. YF, MS, and YY contributed to the literature search, interpretation, writing, and proofreading of the manuscript.

### Conflict of interest statement

The authors declare that the research was conducted in the absence of any commercial or financial relationships that could be construed as a potential conflict of interest.
